# Atraumatic Scapular Spine Fracture: A Rare Injury in a Patient With Rotator Cuff Arthropathy

**DOI:** 10.7759/cureus.11840

**Published:** 2020-12-02

**Authors:** Jonathan McGrath, Jamie Hind, Mark Hamlet, Neil Ashwood

**Affiliations:** 1 Medicine, School of Medicine, University of Leicester, Leicester, GBR; 2 Trauma and Orthopaedics, University Hospitals of Derby and Burton National Health Service Foundation Trust, Derby, GBR

**Keywords:** scapula, fracture, scapular spine fracture, rotator cuff arthropathy

## Abstract

Scapular fractures are uncommon, and the spine of the scapula is a particularly rare site of injury. As a result, our knowledge of these injuries, the management options and the functional outcome is limited. We report a rare case of a scapular spine fracture in a patient with rotator cuff arthropathy with no obvious history of trauma. The pathophysiology behind this is unclear; however, we suggest that a combination of cuff arthropathy, steroid use and chronic cough contributed to it. Reverse total shoulder arthroplasty is commonly used to treat rotator cuff arthropathy, and the effect of scapular spine fractures on surgical outcomes is unknown. It is possible that the deltoid function provides a better indicator of post-operative outcomes.

## Introduction

Scapular fractures make up about 1% of all fractures [1]. They most often occur as a result of a significant force to the scapula and are typically associated with other injuries of the shoulder girdle [1-3]. Fractures of the scapular neck and body are the most common, whilst those of the spine of the scapula make up only around 6% of all scapula fractures [1]. Stress fractures of the scapular spine are especially rare but have been known to occur in individuals with rotator cuff pathology [4]. Here, we report a rare case of a non-union of an atraumatic scapular spine fracture in a patient requiring reverse total shoulder arthroplasty for severe rheumatoid arthritis. In this report, we discuss the possible outcome of reverse total shoulder arthroplasty in the context of non-union of the scapular spine.

This article was previously presented as a poster at the British Association of Clinical Anatomists (BACA) Beat 2 on September 9, 2020.

## Case presentation

A 69-year-old retired dental nurse was referred to the orthopaedic department with right-sided shoulder pain. Her past medical history included chronic obstructive pulmonary disease, bibasal bronchiectasis with kyphoscoliosis, left hemi-diaphragmatic palsy and rheumatoid arthritis. She had vertebral disc protrusion at T12/L1 and L4/L5, which was managed with spinal decompression surgery. Her rheumatoid arthritis was treated with rituximab and prednisolone, as well as analgesia and physiotherapy. Despite this, she had significant pain in her right shoulder. Her erythrocyte sedimentation rate was 1 mm/hr and C-reactive protein was less than 5 mg/L, giving her a Disease Activity Score 28 (DAS28) of 3.37. She had a dual-energy X-ray absorptiometry scan that showed a T-score at the lumbar spine of +0.0 and neck of the femur at -1.3. 

**Figure 1:**
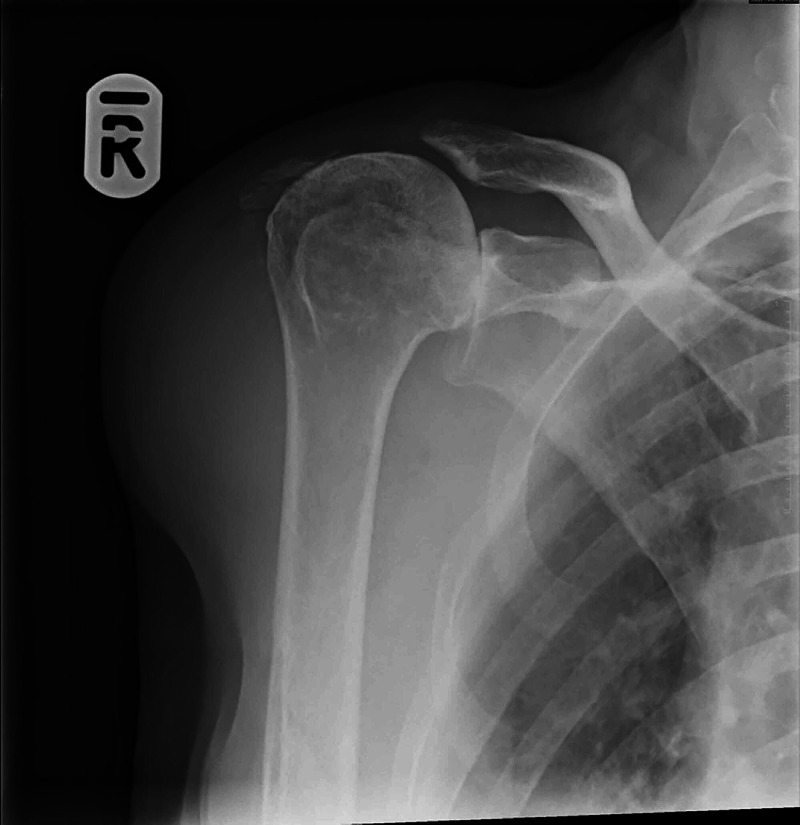
Radiograph of the right shoulder demonstrating superior migration of the humeral head and erosion of the acromion process with destruction of the acromioclavicular joint

**Figure 2:**
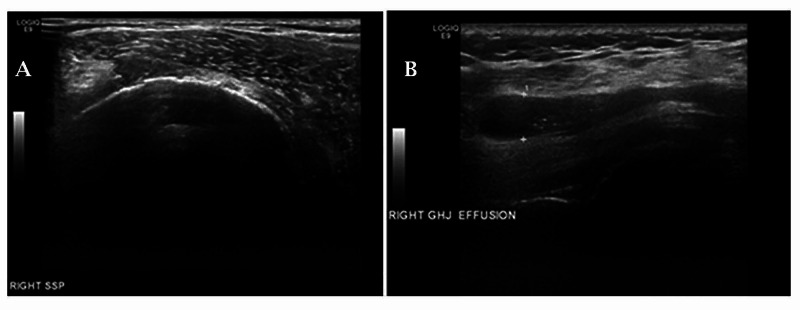
Ultrasound scan of the right shoulder A. Bare humeral head suggestive of rotator cuff tear; B. Effusion of the glenohumeral joint

An X-ray of her shoulder was performed, which demonstrated marked superior migration of the humeral head within the glenohumeral joint and complete destruction of the right acromioclavicular joint (Figure 1). An ultrasound scan demonstrated a muscle tear in her right shoulder (Figure 2A) and a large effusion (Figure 2B). Imaging and examination findings were suggestive of either Milwaukee shoulder or cuff arthropathy.

**Figure 3:**
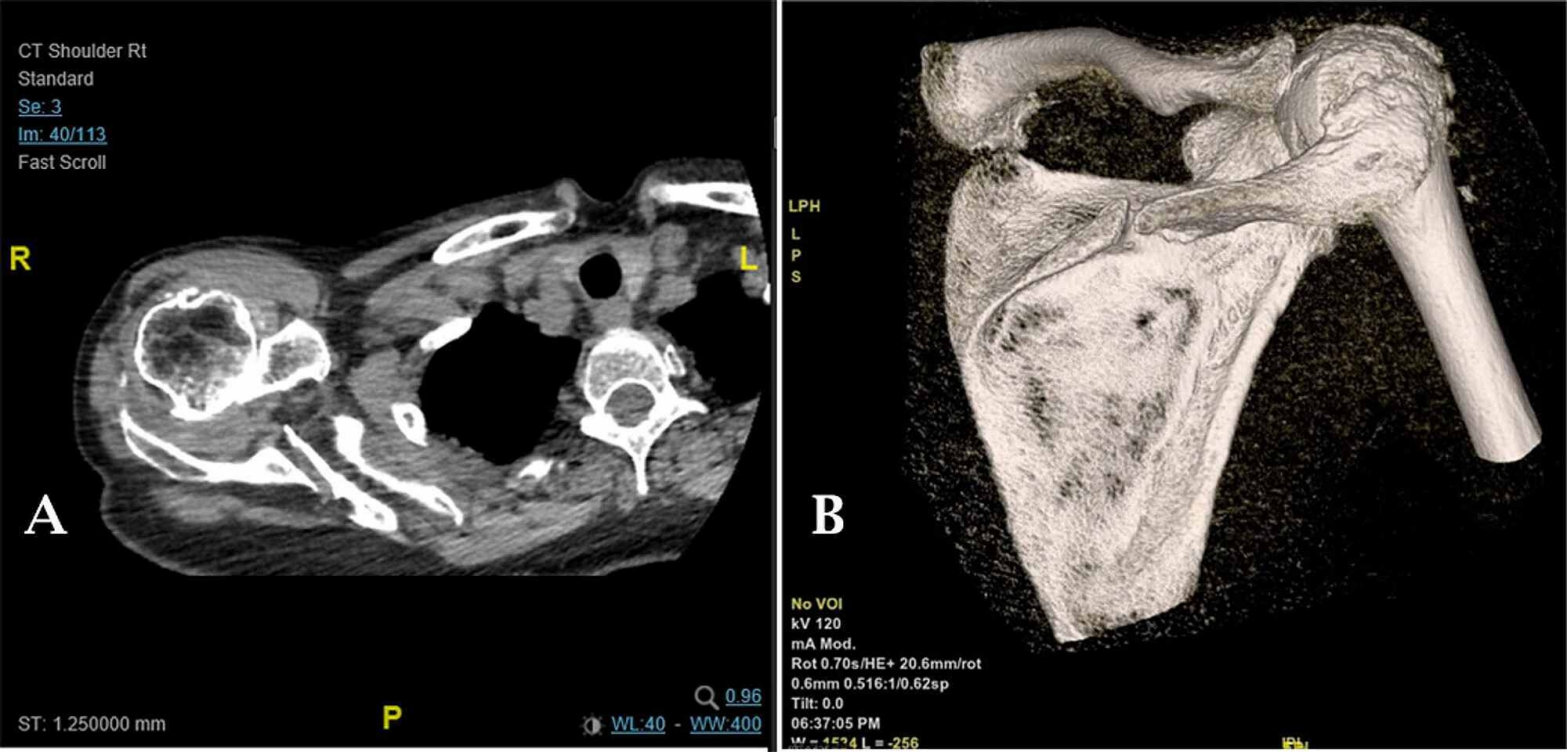
CT images of the right shoulder highlighting a fracture to the spine of the scapula A. Axial CT scan; B. Three-dimensional reconstruction of the CT scan CT: computed tomography

On examination in the orthopaedic clinic, there was swelling around her right shoulder with associated pain on palpation. Her range of movement was severely restricted, and flexion of her right shoulder was limited to 40 degrees. Her deltoid function appeared intact. The suprascapular nerve was also intact and she had functioning supraspinatus and infraspinatus muscles. A computed tomography (CT) scan was performed to establish whether or not reverse total shoulder arthroplasty was suitable. The CT highlighted the severe rotator cuff arthropathy and erosion of the acromion with a calculated glenoid bone stock at 2.8 cm with neutral glenoid version. Interestingly, the CT scan also demonstrated a non-union of a fracture to the medial third of the spine of her scapula (Figure 3). There was no history of trauma.

It appeared fairly firmly adherent with scar tissue with a functioning deltoid, so reverse total shoulder arthroplasty did seem feasible despite the unknown outcome as a result of the non-union of the scapular spine fracture and the erosion of the acromion. Unfortunately, during her pre-operative assessment, it was noted that her bronchiectasis was poorly controlled, so her operation was deferred. Her respiratory condition continued to worsen, and she died eight months later.

## Discussion

Rotator cuff arthropathy can alter the kinematics of the glenohumeral joint, thereby placing increased stress on the scapula [5]. As seen in the plain radiograph of our patient (Figure 1), the humeral head can migrate superiorly following rotator cuff tears, causing an increased strain on the acromion and the scapular spine [6]. The trapezius and deltoid muscles may also alter their activity to compensate for this, further increasing the strain on the scapular spine [4,6-7]. Additionally, it has been postulated that chronic cough can cause trauma to the scapula due to the increased stress placed on the bone by the serratus anterior and rhomboid major muscles [8]. To add to this, our patient’s use of prednisolone causing osteopenia may have further predisposed her to this injury [6-7]. It may well be that our patient’s rotator cuff arthropathy, combined with oral steroid use and chronic obstructive airways disease, culminated in this atraumatic scapular spine fracture.

Whilst there have been good results for both the conservative and surgical management of scapular injuries, there is little in the literature on how these may affect the outcomes of reverse total shoulder arthroplasty [4]. Studies have suggested that pre-operative fractures of the acromion or scapular spine do not affect the outcome of reverse total shoulder arthroplasty, possibly due to the ability of the deltoid to compensate for these fractures through its attachment to the clavicle [9-10]. However, in each of these studies, only one of the patients had a pre-operative scapular spine fracture [9-10]. In cases of rotator cuff arthropathy, reverse total shoulder arthroplasty could actually be beneficial for fractures of the scapular spine by reversing the forces responsible for the stress on the scapular spine. However, there is the potential that this could in fact have a detrimental effect by putting increased stress on the scapular spine, just in the opposite direction [4,9]. It is, therefore, difficult to predict the impact a scapular spine fracture would have on the functional outcome of reverse total shoulder arthroplasty or erosion of the acromion for that matter. It is likely that deltoid function has a higher predictive value on post-operative function, particularly in the presence of rotator cuff arthropathy, in which case, a non-union of a scapular spine fracture is unlikely to affect the outcome.

## Conclusions

Scapular fractures are rare, particularly those of the spine of the scapular. When these fractures do occur, it is often following direct trauma and associated with multiple severe injuries. However, stress fractures of the scapular spine are also a rare complication of rotator cuff arthropathy. Scapular spine fractures are unlikely to affect the outcome of reverse total shoulder arthroplasty for cuff arthropathy, with the deltoid function a likely better indicator of the outcome.
